# Electric field variations across DLPFC targeting methods in TMS therapy for Alzheimer’s disease

**DOI:** 10.1016/j.nicl.2025.103847

**Published:** 2025-07-17

**Authors:** Nianshuang Wu, Yuxuan Shao, Zhen Wu, Shuxiang Zhu, Penghao Wang, Ziyan Zhu, Cheng Zhang, Changzhe Wu, Xiaolin Huo, Hua Lin, Guanghao Zhang

**Affiliations:** a Beijing Key Laboratory of Bioelectromagnetism, Institute of Electrical Engineering, Chinese Academy of Sciences, Beijing 100190, China; b School of Electronic, Electrical and Communication Engineering, University of Chinese Academy of Sciences, Beijing 100049, China; c Department of Neurology, Xuanwu Hospital of Capital Medical University, Beijing 100053, China

**Keywords:** Alzheimer’s disease, Transcranial magnetic stimulation, Targeting, Electric field, Dorsolateral prefrontal cortex

## Abstract

**Background:**

The dorsolateral prefrontal cortex (DLPFC) is crucial for cognitive control and a primary target for transcranial magnetic stimulation (TMS) in Alzheimer’s disease (AD). However, understanding the distribution of TMS-induced electric field (E-field) across different targeting methods remains limited, as does its relationship to therapeutic outcomes.

**Objective:**

This study assesses differences in TMS-induced E-field using functional versus anatomical targeting methods for DLPFC stimulation.

**Methods:**

Functional and anatomical targets were identified in 30 (11 M/19F) AD patients and 30 (13 M/17F) age-matched healthy controls (HCs) using T1 and fMRI data. E-field characteristics, including magnitude (E_ROI_) and normal component (E_⊥_), were calculated via SimNIBS software for comparisons across stimulation targets.

**Results:**

Functional targeting showed greater spatial dispersion compared to anatomical targeting in both groups. Significant E-field differences were observed between the functional target and adjacent anatomical regions when the coil was positioned over the functional target in both groups. Optimal coil orientation exhibited directional specificity: parallel alignment with the LOI E-field produced higher field intensity in the functional target compared to the anatomical target (AD patients: *P* < 0.001; HCs: *P* = 0.052), while perpendicular orientation maintained functional stability with reduced anatomical interference (both groups: *P* < 0.001). And significant variations in E-field ratios were observed across coil orientations.

**Conclusion:**

This study reveals key E-field disparities across DLPFC targeting approaches and establishes coil orientation optimization as a critical strategy to improve TMS precision, offering actionable insights for developing personalized protocols in AD therapy that may enhance treatment efficacy while minimizing adverse effects.

## Highlights

• The spatial dispersion of functional targets is 2.63 times that of anatomical targets.
• E-field at the functional target is significantly higher than that at nearby anatomical target (*P* < 0.001), when the coil is positioned above functional target.
• Coil orientation selectively modulates E-fields. With the handle perpendicular to the anatomical-functional target axis, functional target E-field was maintained while anatomical target E-field was suppressed.

## 1 Introduction

Repetitive transcranial magnetic stimulation (rTMS) has shown potential for cognitive enhancement in Alzheimer's disease (AD) patients (Canter et al., 2016, Weiler et al., 2020, Wu et al., 2022). As a focal brain stimulation technique, rTMS relies on the precise targeting of specific brain areas, where its efficacy is largely determined by the accuracy of the chosen stimulation target and the methods used to identify it (Fox et al., 2012, Herbsman et al., 2009). Central to this process is the generation of electric field (E-field) distributions within the brain, which are critical in modulating neural activity at the target site(Argyelan et al., 2019, Deng et al., 2022, Zanto et al., 2021). The majority of clinical trials and therapeutic have targeted the dorsolateral prefrontal cortex (DLPFC), typically on the left (e.g., Ref. (O'Reardon et al., 2007)), but sometimes on the right (Isenberg et al., 2005), or bilaterally (Berlim et al., 2013, Blumberger et al., 2012). Current targeting paradigms use three primary neuroimaging approaches: functional magnetic resonance imaging (fMRI), structural anatomical atlases, and EEG-based localization. Despite their widespread application, these methods do not sufficiently integrate the E-field distribution that arises from external stimulation, which can lead to unintended effects on adjacent brain regions, potentially compromising the specificity and efficacy of the treatment.

The DLPFC is functionally defined as the cortical area involved in cognitive control and is classically attributed to anatomical Brodmann areas 9 and 46 (BA 9/46) (Cieslik et al., 2013). Unlike primary sensorimotor and visual cortices, which can be mapped using objective electrophysiological biomarkers such as motor evoked potentials and phosphene thresholds, the DLPFC lacks reliable physiological biomarkers. The absence of such markers complicates the accurate localization of this area for rTMS. The motor cortex, for instance, is more easily identified using structural MRI landmarks (Boroojerdi et al., 1999, Yousry et al., 1997), and the functional motor hotspot is defined by scalp locations eliciting MEPs with 50 % probability (Rossini et al., 1994). However, these techniques are not applicable to higher-order cognitive regions like the DLPFC, thus challenging accurate targeting for rTMS. One of the earliest and most widely used methods for locating the DLPFC is the so-called “5 cm rule” (Pascual-Leone et al., 1996). While relatively simple, this method suffers from reduced accuracy and, when compared to MRI, often locates a site outside the DLPFC proper (George et al., 2010, Herwig et al., 2001). A more refined approach uses the international 10–20 EEG system, where the F3 and F4 electrodes are often used as approximate locations for the left and right DLPFC (Guse et al., 2013, Herwig et al., 2003). Additionally, research identified stereotaxic coordinates for an ‘optimized’ DLPFC-rTMS target (at Montreal Neurological Institute coordinates, X: −38, Y: +44, Z: +26) based on negative correlation of its resting-state functional connectivity to a seed region in the subgenual cingulate target. Emerging network-based approaches use deep brain stimulation (DBS) targets as functional connectivity seeds to identify optimal cortical modulation sites (Fox et al., 2014, Ji et al., 2021), but these still do not fully address the potential impact of E-field spread on surrounding regions (Lozano et al., 2008). These limitations underscore the need for more accurate and comprehensive targeting methods that integrate E-field analysis.

The role of the E-field and coil orientation in rTMS efficacy is increasingly recognized, as it directly influences neural activity not only at the target site but also potentially in adjacent regions (Cerins et al., 2024). While current rTMS targeting methods focus on functional or anatomical coordinates, they fail to fully account for how the E-field distribution can inadvertently activate neighboring areas, thus potentially altering the overall brain network function. This oversight is particularly problematic when considering the complexities of neurodegenerative diseases like AD, where structural changes such as cortical atrophy alter the scalp-to-cortex distance (SCD), complicating E-field delivery and efficacy. Such anatomical changes may explain the reduced effectiveness of rTMS in advanced AD cases despite adherence to standardized protocols. Emerging evidence suggests that E-field characteristics, such as magnitude, are crucial for modulating the therapeutic effects of rTMS. For example, the normal E-field component, rather than the tangential components, correlates with antidepressant outcomes (Zhang et al., 2022). Additionally, therapeutic mechanisms of rTMS appear to involve distributed network modulation rather than direct stimulation of the cortical target (Hua et al., 2024). This highlights the importance of considering E-field properties in rTMS protocols. However, the existing methods predominantly rely on single-modality targeting, which does not sufficiently account for individual differences in neuroanatomy that lead to variable E-field distributions (Vöröslakos et al., 2018). In AD neurodegeneration, cortical atrophy increases SCD through sulcal widening and gyral thinning, potentially explaining diminished rTMS efficacy in advanced cases despite protocol adherence (Balderston et al., 2020).

This study aims to address these gaps by comparing the spatial E-field characteristics of three DLPFC targeting methods: functional targets, anatomical targets, and F3-based targets. Additionally, the research seeks to clarify the influence of functional target stimulation on adjacent brain regions and improve focusing accuracy. Utilizing individualized head models, we analyzed spatial distances and E-field distributions across the targets. We also developed an optimization strategy to improve focality of functional targeting methods to reduce the influence of neighboring brain regions. Comparisons between AD patients and healthy controls (HCs) were systematically performed. The results of this investigation will advance the personalization of rTMS therapy and improve its clinical outcomes in neurodegenerative disorders.

## 2 Methods

### 2.1 Participants

We recruited 30 patients diagnosed with mild cognitive impairment (MCI) due to AD from the outpatient clinic service of Xuanwu Hospital, Capital Medical University, Beijing, China. All participants provided written informed consent prior to participation. Diagnoses for all patients were made by two trained senior neurologists following a detailed consultation. This group consisted of 11 male and 19 female patients, ages 55–79 (mean 68.70 ± SD 6.86). In this study, 30 patients underwent rTMS targeting the left DLPFC from August 2022 to August 2023. The inclusion criteria were as follows: (i) all participants met 2011 NIA-AA criteria for MCI due to Alzheimer’s disease; (ii) right-handed male or female patients aged 55–80 years; (iii) Clinical Dementia Rating (CDR) point of 0.5; (iv) Mini-Mental State Examination (MMSE) ≥ 20; and (v) stable donepezil dosage for at least three months prior to and during the aiTBS intervention. The exclusion criteria were as follows: (i) presence of non-AD pathologies, other neurological or severe systemic diseases; (ii) history of seizures or first-degree relatives with seizures; (iii) current benzodiazepine use or substance abuse history; (iv) focal brain lesions on MRI (T1 or T2); and (v) non-MRI compatible metallic implants (e.g., pacemakers, deep brain stimulators). See Supplementary Materials (Section 1) for details. This study is registered in the Chinese Clinical Trial Registry, number ChiCTR2200062564, with additional data from 15 AD cases available in the Supplementary Materials (Section 2). Additionally, we included 30 age-matched HCs selected from the public dataset (https://openneuro.org/datasets/ds005270/versions/1.0.0, Supplementary Materials Section 3) (Rieck. et al., 2024).

### 2.2 MRI acquisition

All structural and resting-state functional MRI (rs-fMRI) data were collected by a Siemens 3.0T MRI system (Siemens, Erlangen, Germany). The main parameters for magnetization-prepared rapid gradient echo (MPRAGE) images were: repetition time (TR) = 2530 ms, echo time (TE) = 2.98 ms, inversion time (TI) = 1100 ms, flip angle (FA) = 7°, matrix size = 256 × 256, slices = 192, voxel size = 1 × 1 × 1 mm^3^. Rs-fMRI data were collected over three consecutive sessions using a T2-weighted gradient-echo echo planar imaging (EPI) sequence sensitive to blood oxygenation level-dependent (BOLD) contrast. Functional images were acquired with: TR = 2000 ms, TE = 30 ms, field of view (FOV) = 220 × 220 mm^2^, FA = 90°, acquisition matrix = 64 × 64, yielding 32 interleaved axial slices with 0 mm gap (3.4 × 3.4 × 3.5 mm^3^ voxel size). Each rs-fMRI session lasted 12 min 0 s (360 volumes), during which participants were instructed to keep their eyes closed and remain motionless.

### 2.3 E-field modeling

**Fig. 1:**
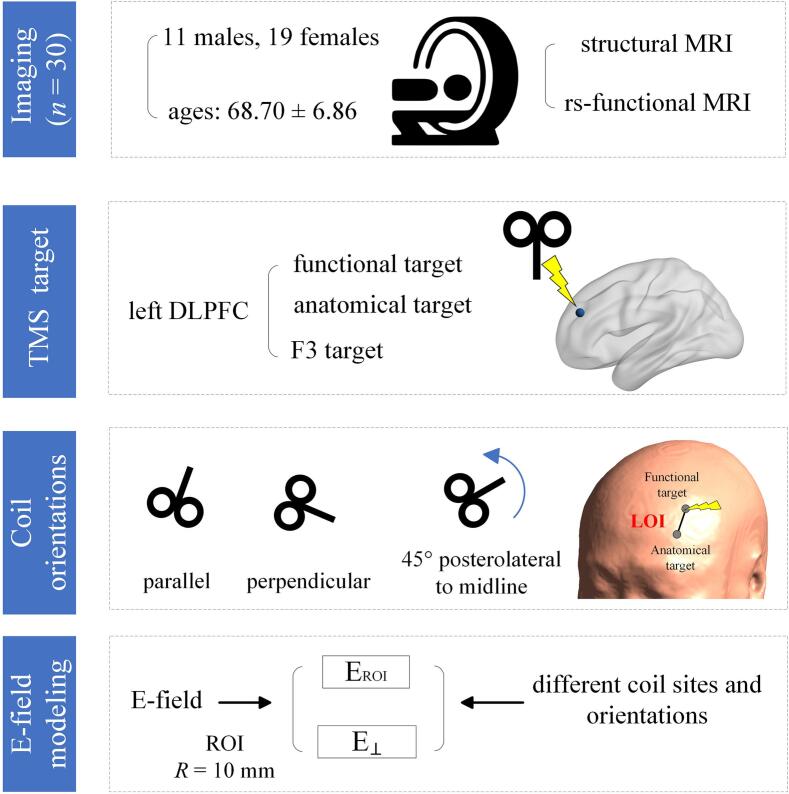
Overall study design. MRI data from 30 patients were used to locate the target site, specifically the left DLPFC. The target localization was performed using three methods: 1) the functional target, 2) the anatomical target, and 3) the F3 target. Subsequently, an E-field modeling was conducted with a 10-mm radius for the ROI, focusing on the distributions of E_ROI_ and E_⊥_. A LOI was defined as the line connecting the functional and anatomical targets. The coil orientation is categorized into three cases: 1) parallel to the LOI; 2) perpendicular to the LOI; and 3) the initial orientation has the coil handle pointing posterolaterally at a 45° to the mid-sagittal plane, subsequently rotated 150°.

The study concentrated on the left DLPFC as the target region, with the workflow depicted in Fig. 1. All participants underwent MRI data processing through SimNIBS software (v3.2.5, https://www.simnibs.org). The T1-weighted images were segmented using the 'mri2mesh' command to generate a realistic brain model that simulates the E-field distribution. The model incorporated multiple tissue layers, including the scalp, skull, cerebrospinal fluid (CSF), gray matter (GM), and white matter (WM), with their respective conductivity values set as default settings in the software: 0.465 S/m for scalp, 0.010 S/m for skull, 1.654 S/m for CSF, 0.275 S/m for GM, and 0.126 S/m for WM. Three targeting methods were used: functional, anatomical, and F3-based (Herwig et al., 2003).

#### 2.3.1 Coil positions and orientations

Determination of functional target site.

The determination of functional target site is based on rs-fMRI data and functional connectivity analysis, utilizing the TMStarget software (https://github.com/jigongjun/Neuroimaging-and-neuromodulation). First, the rs-fMRI data is preprocessed (detailed steps provided in the Supplementary Materials Section 4) to remove noise and standardize the data. The left hippocampal formation (MNI coordinates: [-26, –22, −12]) served as the seed region for functional targeting (Chand et al., 2018, Naia et al., 2023, Zhao et al., 2020). Subsequently, functional targets are extracted through seed-based connectivity analysis. These functional connectivity targets are then mapped to the individual subject's anatomical space and optimized in position to ensure that the targets are located at the nearest cortical surface, thereby enhancing the accuracy of the targeting process.

Determination of anatomical target site.

The determination of the anatomical target site is based on T1-weighted MRI data, processed using the SimNIBS software. Segmented T1 data enables the mapping of predefined MNI coordinates (−38, 44, 26) (Mir-Moghtadaei et al., 2015) to the individual subject's space, facilitating the identification of specific anatomical regions in the brain. Through optimization of localization steps, the target points are adjusted to align with the nearest cortical surface, thereby ensuring precise anatomical positioning accuracy.

Determination of EEG-F3 target site.

The EEG-F3 target site was identified by integrating an individualized head model constructed using the SimNIBS software. Based on the individual’s T1-weighted MRI data, a personalized head model was created. The spatial information of the EEG scalp electrode F3 was then obtained. Through coordinate mapping, the position of the F3 electrode was optimized to ensure it was aligned with the nearest cortical surface, thereby achieving accurate localization of the EEG-F3 target site.

Different coil placement configurations.

Coils were placed tangentially to the skull, with the handle pointing posterolaterally at a 45° angle to the mid-sagittal plane. The junction point of the figure-8 coil was positioned at the scalp point closest to the intracerebral target (Wu et al., 2022). This position and orientation were defined as the initial orientation (0°) for this study. The line connecting the coil center and the intracranial target was defined as the rotation axis. Starting from 0°, the coil was rotated counterclockwise around the rotation axis in 30° increments up to 150°. Due to the symmetry of the stimulation coil, E-field values for coil orientations from −180° to −30° were calculated by mirroring the corresponding orientations from the first half of the rotation (0° to 150°). Additionally, we investigated two specific coil orientations: one with the coil handle parallel to the line of interest (LOI), and another perpendicular to the LOI. The LOI was defined as the line connecting the anatomical target and the functional target, and these two placement conditions helped assess the specific effects of coil orientation on E-field distribution.

#### 2.3.2 Stimulation strength

The stimulation strength is determined by the d*i*/d*t* parameter in SimNIBS software, which represents the rate of current change. For the M1 target (MNI coordinate: −34.19, −14.33, 66.83), the d*i*/d*t* value is set to 1 A/μs, whereas for the left DLPFC target, the d*i*/d*t* values are adjusted to 0.8 A/μs, corresponding to 80 % of the resting motor threshold (rMT).

#### 2.3.3 E-field analyses

The ROI was defined as a sphere of 10 mm in radius, centered at the cortical surface location with minimal SCD. This SCD metric was calculated as the Euclidean distance along the scalp surface normal vector between the scalp stimulation site and its nearest cortical projection point, ensuring orthogonal measurement relative to the local scalp curvature. Within each ROI, the number of voxels was calculated. The normalized E-field strength (E_ROI_) within the ROI was computed by averaging the E-field strength across all voxels and normalizing it by dividing by the E-field strength at the initial coil orientation's M1 E-field. Particular emphasis was placed on the normal component of the E-field (E_⊥_), which is perpendicular to the cortical surface, based on prior studies demonstrating its critical role in effective neural activation (Zhang et al., 2022). With the coil positioned stably, E-field were measured at three target sites: the functional target, anatomical target, and F3 target. For instance, when the coil was oriented at the functional target position, E-field was calculated and analyzed at all three sites. This methodology was consistently applied to other target sites.

### 2.4 Statistical analyses

The statistical analyses were mainly completed through SPSS Version 26 software (IBM Corp. in Armonk, NY) and MATLAB (v.8.5, R2015a). Unless specified otherwise, a *P* < 0.05 was considered statistically significant. To analyze the spatial distribution characteristics and dispersion of three types of target points, we first calculated the centroid of the targets, which is the mean point of all target coordinates. Then, for each target point, the distance to the centroid was calculated. Finally, the average of these distances was computed to assess the dispersion of the target point distribution. The cortical points of the subjects were mapped to the MNI space, and the distances between all target pairs were calculated. ANOVA was performed to assess spatial differences and E-field between the three targets, followed by post-hoc LSD analysis. Additionally, a T-test was used to examine the differences in E-fields between the anatomical and functional targets when the coil was aligned parallel and perpendicular. The ratio of functional to anatomical E-fields across different coil orientations was also analyzed using ANOVA, with post-hoc LSD analysis to evaluate specific group comparisons.

## 3 Results

### 3.1 Distances and voxel values across different targets

**Fig. 2:**
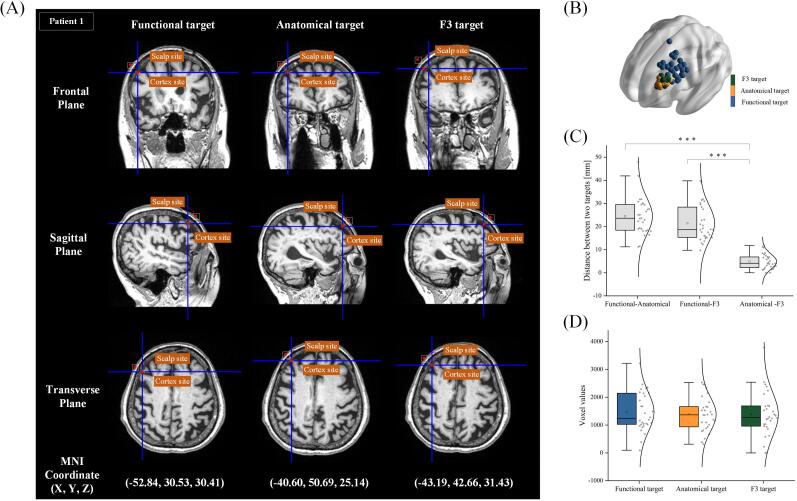
Spatial and voxel differences. A. Using Patient 1 as an example, the spatial distances between the cortical points and scalp points for the three targets were presented across three planes, with the MNI coordinates indicating each target's cortical points. B. The three target sites for 30 patients were mapped to MNI space, showing that the anatomical and F3 targets had more concentrated distributions and shorter spatial distances, whereas the functional target exhibited a more dispersed distribution. C. The distances from the among the three targets in all patients were compared. No significant difference was observed between the functional-anatomical and functional-F3 targets, while other comparisons showed significant differences (*P* < 0.001). Mean (□) and median (—) values are indicated. D. Voxel distributions of the ROI on the cortex for all three targets were analyzed, revealing no significant differences between them.

Fig. 2A illustrates the distribution of target points identified using three localization methods across different planes, with patient 1 serving as an example. Anatomical targets in patient 1 showed the greatest SCD (12.97 mm), followed by F3 (12.92 mm) and functional targets (12.85 mm). The mean SCD across patients was 14.76 ± 2.41 mm for functional targets, followed by 13.13 ± 1.74 mm for F3 and 12.75 ± 1.62 mm for anatomical targets.

Spatial dispersion analysis revealed a hierarchical distribution pattern among the three target groups (Fig. 2B, Table S1, ANOVA: *F* = 37.60, *P* < 0.001, η^2^ = 0.46). Functional targets demonstrated significantly greater variability with a mean radial distance of 13.61 ± 6.82 mm, 2.63-fold higher than anatomical targets (5.17 ± 2.66 mm, *P* < 0.001). F3 targets exhibited the most compact spatial configuration (4.59 ± 2.75 mm), showing a 66.27 % reduction in dispersion compared to functional targets (*P* < 0.001), though no significant difference was observed versus anatomical targets (11.2 % reduction, *P* = 0.62).

Analysis revealed significant variations in inter-target spatial distances (*F* = 72.13, *P* < 0.001, η^2^ = 0.62; Fig. 2C, Table S2). The anatomical-F3 target pair exhibited the closest spatial proximity (4.81 ± 3.28 mm), significantly shorter than both functional-anatomical (24.45 ± 8.04 mm, *P* < 0.001) and functional-F3 distances (21.40 ± 8.00 mm; *P* < 0.001). Notably, the functional-anatomical distance showed marginal significance compared to functional-F3 separation (*P* = 0.087). These variations in spatial positions may potentially influence intracranial E-field distributions.

Volumes analysis demonstrated comparable ROI brain volumes across targets (Fig. 2D, Table S3): functional (1469.70 mm^3^ ± 705.12 mm^3^), anatomical (1385.07 mm^3^ ± 614.93 mm^3^), and F3 (1383.73 mm^3^ ± 795.79 mm^3^), without statistical significance (ANOVA *F* = 0.14, *P* = 0.87, η^2^ = 0.003). Post-hoc LSD confirmed non-significant pairwise comparisons (functional-anatomical: *P* = 0.65; functional-F3: *P* = 0.64; anatomical-F3: *P* = 0.99).

We also calculated the SCD and voxel values across different targets in HCs (Table S12). Notably, functional targets demonstrated significantly greater spatial variability than anatomical targets in HCs (mean radial distance: 19.56 ± 7.03 mm vs. 3.70 ± 2.54 mm; *t* = 17.45, *P* < 0.001, *d* = 3.00, effect size = 0.83), consistent with the result in AD patients.

### 3.2 Comparison of E-field across different targets at the same coil orientation

**Fig. 3:**
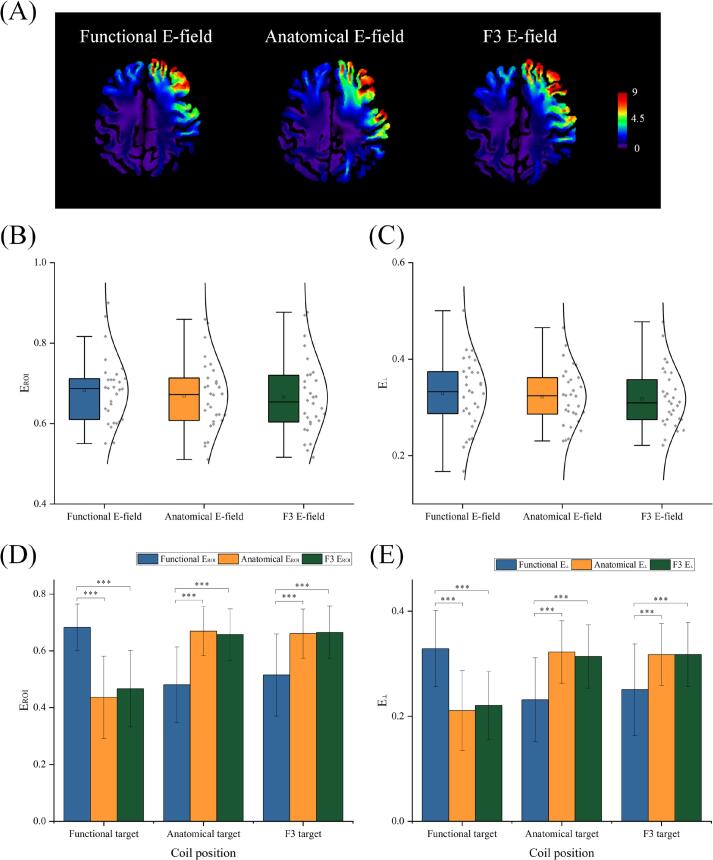
E-field differences. A. Normalized E-field distributions at the initial orientation are illustrated using data from patient 1 at a d*i*/d*t* of 1 A/μs, when the coil was placed at three different target sites. B-C. When the coil was placed at the three target sites, the corresponding E-field values at each target (E_ROI_, B and E_⊥_, C) were measured. No significant differences were observed between the E-fields at the three target sites for either E_ROI_ or E_⊥_. D-E. When the coil was positioned at the target sites, the E-field differences between the target and adjacent sites were analyzed. Regardless of whether the coil was placed, or whether E_ROI_ (D) or E_⊥_ (E) was measured, no significant differences were found between the anatomical and F3 targets (*P* > 0.05).

Using Patient 1 as an example, the E-field distributions under different stimulation targets are shown (Fig. 3A). When the coil was aligned with the functional target, (Fig. 3B for E_ROI_ and 3C for E_⊥_), the E-field was 0.68 ± 0.082 for E_ROI_ and 0.33 ± 0.072 for E_⊥_. For the anatomical target, the values were 0.67 ± 0.086 for E_ROI_ and 0.32 ± 0.059 for E_⊥_. When the coil was aligned with the F3 target, the E-field values were 0.66 ± 0.093 for E_ROI_ and 0.32 ± 0.061 for E_⊥_. The results showed that there were no significant differences (*P* > 0.05, Table S4) in the E-field among these three targets. Although there were spatial differences between the anatomical and functional targets and differences in the coil-to-target distance, the E-field directly below the coil did not show any significant variation.

Subsequently, we compared the E-field at the neighboring targets for each coil alignment (Fig. 3D for E_ROI_ and 3E for E_⊥_). When the coil was aligned with the functional target, the E_ROI_ at the anatomical target and F3 target were 0.44 ± 0.14 and 0.47 ± 0.13 respectively. The corresponding E_⊥_ at the anatomical target and F3 target were 0.21 ± 0.076 and 0.22 ± 0.065. When the coil was aligned with the anatomical target, the E_ROI_ values at the functional and F3 targets were 0.48 ± 0.13 and 0.66 ± 0.090 respectively. For the E_⊥_, the values at the functional and F3 targets were 0.23 ± 0.080 and 0.31 ± 0.060 respectively. Lastly, when the coil was aligned with the F3 target, the E_ROI_ values at the functional and anatomical targets were 0.51 ± 0.14 and 0.66 ± 0.086, respectively. The E_⊥_ values at the functional and anatomical targets were 0.25 ± 0.087 and 0.32 ± 0.059, respectively.

The results indicated significant differences (*P* < 0.001, Table S5) in the E-field intensities between the functional and anatomical targets, as well as between the functional and F3 targets, regardless of the coil position. In contrast, no significant differences were observed between the E-fields of the anatomical and F3 targets (*P* > 0.05, Table S5). Consequently, the following analyses will focus primarily on the functional and anatomical targets.

The E-field distribution patterns in HCs showed both similarities and differences compared to AD patients. Consistent with AD findings, HCs demonstrated: no significant E-field differences directly beneath the coil across different targets (Table S13), and significantly different E-field between functional targets and neighboring anatomical targets when the coil was positioned over functional targets (*P* < 0.05, Table S14). However, a notable divergence emerged when examining anatomical target alignment: unlike in AD patients where differences were observed, HCs showed no significant difference (*P* > 0.05, Table S14) in E-field between anatomical targets and their adjacent functional targets.

### 3.3 The impact of orthogonal coil positioning on target sites

#### 3.3.1 Dual-target E-field maximization strategy (coil handle parallel to the LOI)

**Fig. 4:**
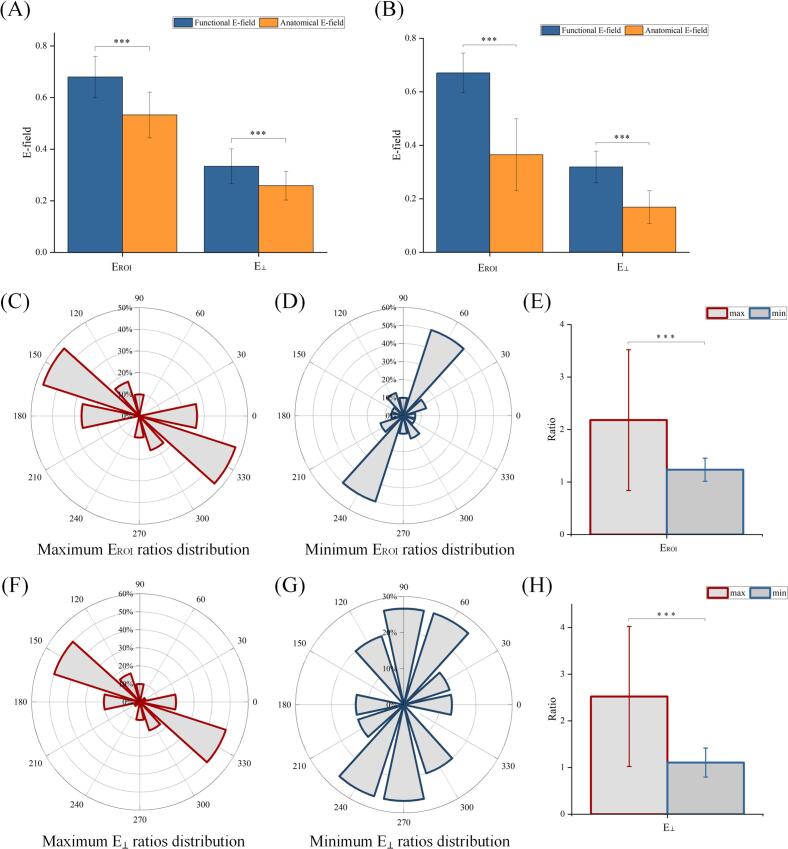
Coil handle parallel or perpendicular to the LOI. A. When the coil handle was aligned parallel to the LOI, significant differences were observed between the functional and anatomical targets for both E_ROI_ and E_⊥_. B. When the coil handle was aligned perpendicular to the LOI, significant differences were also observed between the functional and anatomical targets for both E_ROI_ and E_⊥_. C-H. Effects of coil orientation on stimulation targets. When the coil handle was rotated, the E_ROI_ (C-E) and E_⊥_ (F-H) ratios between functional and anatomical targets were measured across orientations. E_ROI_ ratios showed significant differences between its maximum and minimum values (E), with Fig. 4C-D displaying the percentage of patients achieving maximal (C) and minimal (D) E-field ratios per orientation. Similarly, E_⊥_ ratios showed significant differences between their maximum and minimum values (H), where percentage of patients achieving maximal (F) and minimal (G) E-field ratios per orientation.

When the coil handle was aligned parallel to the LOI, the E-field distribution for the E_ROI_ at the functional target was 0.68 ± 0.064. In contrast, the anatomical target had an E_ROI_ value of 0.55 ± 0.074 (Fig. 4A), resulting in a ratio of 1.24. Statistical analysis using a T-test revealed a significant difference between the two targets (*t* = 6.38, *P* < 0.001, *d* = 1.88, effect size = 0.68). A parallel analysis of the E_⊥_ showed similar spatial specificity. Specifically, the functional target exhibited an E_⊥_ value of 0.34 ± 0.057, while the anatomical target showed a value of 0.27 ± 0.048 (Fig. 4A), resulting in a ratio of 1.26. A T-test further confirmed this difference (*t* = 4.54, *P* < 0.001, *d* = 1.33, effect size = 0.55).

#### 3.3.2 Target-specific activation strategy (coil handle perpendicular to the LOI)

To achieve functional target-specific activation with concurrent anatomical target suppression, we implemented orthogonal coil positioning by aligning the handle perpendicular to the LOI. This configuration preserved the efficacy of the functional target while significantly reducing the activation of the anatomical target. For the E_ROI_, the functional target intensity remained stable at 0.66 ± 0.070 (−2.94 % compared to parallel alignment of 0.68 ± 0.064), whereas the anatomical target E_ROI_ showed a marked reduction to 0.39 ± 0.11 (−29.09 % compared to parallel alignment of 0.55 ± 0.074, Fig. 4B). The ratio between these values was 1.69, with a T-test showing a significant difference (*t* = 9.94, *P* < 0.001, *d* = 2.93, effect size = 0.83).

A parallel analysis of the E_⊥_ revealed similar modulation patterns: the functional target intensity was preserved at 0.31 ± 0.057 (−8.82 % compared to parallel alignment of 0.34 ± 0.057), while the anatomical target suppression was greater, with an E_⊥_ value of 0.18 ± 0.049 (–33.33 % compared to parallel alignment of 0.27 ± 0.048, Fig. 4B). The ratio between these values was 1.72, with a T-test confirming this significant difference (*t* = 8.16, *P* < 0.001, *d* = 2.45, effect size = 0.77).

A comparison of dual-target stimulation outcomes revealed both concordant and discordant results between HCs and AD patients (Table S15). With parallel handle alignment (dual-target maximization), the E_⊥_ difference between targets showed *P* = 0.052 (compared to *P* < 0.001 in AD patients). However, perpendicular handle orientation (target-specific activation) maintained significant E-field differentiation between targets (*P* < 0.001) in both groups.

#### 3.3.3 E-field ratios at various orientations for functional and anatomical targets

As the coil rotates from its initial position, it is positioned above the functional target site. At different orientations, we measured the ratio of functional to anatomical E-field (Table S6). In the E_ROI_ ratio analysis (Fig. 4C and 4D), the maximum ratio occurred at 0° in 26.67 % of patients, while 46.67 % of patients showed peak ratios at 150°. Additional peaks were observed at 90° (10.00 %) and 120° (16.67 %). The minimum ratio is observed at 0° (6.67 %), 30° (13.33 %), 60° (50.00 %), 90° (10.00 %), 120° (13.33 %), and 150° (6.67 %). A T-test reveals a significant difference between the maximum (2.18 ± 1.34) and minimum (1.23 ± 0.22) values (*t* = 3.81, *P* < 0.001, *d* = 0.99, effect size = 0.44, Fig. 4E).

Similarly, the ratio of E_⊥_ exhibits the following variations (Fig. 4F and 4G): the maximum ratio is observed at 0° (20.00 %), 30° (3.33 %), 90° (10.00 %),120° (16.67 %), and reaches 50.00 % at 150°. The minimum ratio occurs at 0° and 30° (13.33 %), 60° and 90° (26.67 %), 120° (20.00 %). Statistical analysis demonstrates a significant difference between the maximum (2.52 ± 1.50) and minimum (1.11 ± 0.31) values (*t* = 5.06, *P* < 0.001, *d* = 1.30, effect size = 0.55, Fig. 4H).

HCs and AD patients showed different patterns in how the functional-to-anatomical E-field ratios changed with coil orientation. For E_ROI_ ratios, HCs had maximum ratios spread across multiple orientations (Supplementary Materials Section 3), with the highest at 90° (26.67 %). In contrast, AD patients showed clustered maximum ratios at 150° (46.67 %). Minimum ratios were more evenly distributed in HCs (23.33 % at 150°) compared to AD patients where they concentrated at 60° (50.00 %). Both groups had significant differences between maximum and minimum ratios (HCs: 3.27 ± 2.16 vs 1.41 ± 0.34, t = 4.67, *P* < 0.001, *d* = 1.20, effect size = 0.52).

For E_⊥_ ratios, both groups peaked at 150°, but AD patients had a higher proportion (50.00 % vs 26.67 % in HCs). Minimum ratios clustered at 60° and 90° in AD patients (26.67 % each) versus 120° and 150° in HCs (20.00 % each). Detailed data are available in Supplementary Materials (Section 3). The maximum-minimum differences remained significant in both groups (HCs: 3.67 ± 2.49 vs 1.26 ± 0.46, *t* = 5.21, *P* < 0.001, *d* = 1.35, effect size = 0.56).

## 4 Discussion

This study is the first systematic investigation into the characteristics of the E-field generated by different TMS targeting methods in AD patients, with parallel comparison in HCs. Our quantitative spatial analysis revealed that functional targeting exhibited the highest spread characteristics compared to alternative approaches in both groups. When the coil was positioned directly above the target point, functional and anatomical targeting modalities showed comparable peak E-field intensities (*P* > 0.05) in both patients and HCs. However, adjacent cortical regions exhibited significantly different E-fields in both groups (*P* < 0.05) when the coil is positioned over the functional target, suggesting critical spatial specificity in stimulation patterns. Coil handle orientation relative to the LOI critically determined E-field distribution. Positioning the coil perpendicular to the LOI provides a more effective way to enhance functional targeting while minimizing anatomical interference in both HCs and patients. Additionally, the ratio of E-fields between functional and anatomical targets changed with coil orientation, highlighting the importance of optimizing coil positioning for effective stimulation in AD research. This comprehensive analysis underscores the necessity of precise coil positioning strategies for effective and targeted neurostimulation in AD patients.

While no significant differences emerged in SCD measures between groups consistent with MCI-stage AD cohort (Lu et al., 2021), distinct E-field differences were detected. In HCs, coil positioning at the anatomical target produced no significant E-field difference between its center and adjacent functional regions, whereas alignment with the functional target yielded significant differences. Previous study has illustrated that E-fields stimulated within functional networks tend to propagate along those networks, rather than diffusing randomly (Hua et al., 2024). However, in AD patients, underlying pathologies – including brain atrophy, amyloid-beta plaques, and tau tangles (Jack et al., 2013, Opitz et al., 2011, Pereira et al., 2021) – severely disrupt synaptic connections and network integrity, likely contributing to E-field characteristics contrasting with HCs. Therefore, achieving effective neuromodulation in AD necessitates a greater emphasis on personalization and precision. Targeting the core functional node of the relevant network becomes the preferred strategy, as relying solely on anatomical landmarks may be insufficient due to the compromised network dynamics. Our comparative analysis revealed that positioning the coil perpendicular to the LOI provides a more effective way to enhance functional targeting while minimizing anatomical interference in both patients and HCs.

SCD is a critical parameter in the precise treatment of TMS, particularly important in the localization of the left DLPFC (Lu et al., 2019, Lu et al., 2021). It has been observed that SCD for MCI converters is 14.75 ± 2.15 mm (Lu et al., 2021), while SCD for AD patients is 16.09 ± 2.89 mm (Lu et al., 2019). Age and dementia status exhibited differential effects on SCD in the left DLPFC (Lu et al., 2019). In our study targeting the same region, we conducted the first systematic comparison of SCD under two localization methods: 14.76 ± 2.41 mm for functional targets and 12.75 ± 1.62 mm for anatomical targets. Notably, our spatial analysis revealed significantly greater radial dispersion of functional targets in AD patients (13.61 ± 6.82 mm) compared to anatomical targets. This finding extends previous observations of inter-individual variability in connectivity-guided TMS targeting from depression studies to the AD population (Cash et al., 2021, Kong et al., 2022). Critically, by integrating spatial distance from different localization methods with voxel analysis, we demonstrated systematic spatial discrepancies between functional and anatomical targeting approaches in AD patients, thereby establishing a novel framework for understanding targeting precision in TMS interventions for neurodegenerative disorders.

Previous studies have highlighted the critical role of SCD and connectivity-guided functional targets in determining E-field characteristics (Cash and Zalesky, 2024, Kong et al., 2022, Lu et al., 2021), our age-controlled analysis further confirms that SCD remains significantly correlated with E-field distribution (see Supplementary Materials Section 5). Current therapeutic protocols demonstrate insufficient optimization challenges in differentiating functional-anatomical target interactions. Precise stimulation of functional targets with avoidance of irrelevant network activation is crucial for therapeutic efficacy optimization (Ozdemir et al., 2020). Existing research predominantly focuses on functional target engagement while neglecting the critical evaluation of off-target effects − particularly how E-field influences adjacent anatomical targets during standard stimulation protocols. This knowledge gap becomes clinically consequential given that commonly employed anatomical targets may receive unintended subthreshold stimulation, potentially compromising treatment specificity. The choice of stimulus intensity and intracranial E-field strength substantially impacts rTMS outcome (Turi et al., 2022). Current research confirms that the stimulation intensity for rTMS in treating AD is typically set between 80 % and 110 % of the MT potentially compromising treatment efficacy (Lin et al., 2019, Turi et al., 2021). While established literature confirms that rTMS mediates therapeutic effects through network-level modulation rather than isolated target stimulation (Hua et al., 2024), our study provides novel evidence by elucidating orientation-dependent E-field relationships between functional and anatomical targets. Under conventional parallel orientation (functional/anatomical ratio: 1.24), anatomical regions receive 80.6 % MT stimulation—approaching therapeutic thresholds—revealing how standard protocols may inadvertently activate non-target networks. In contrast, our perpendicular orientation paradigm achieves enhanced network selectivity (ratio: 1.69), maintaining anatomical regions at subtherapeutic levels (59.17 % MT) while ensuring full therapeutic intensity at functional targets. This dual-target modulation capability represents a paradigm shift from traditional single-intensity approaches, enabling simultaneous precision control over network node activation and circuit suppression.

Our findings align with previous studies demonstrating the critical influence of coil orientation on TMS stimulation outcomes (Balderston et al., 2020). The observed variations in E-field ratios across different coil rotations confirm earlier reports that conventional coil positioning may not be optimal for DLPFC targeting (Cerins et al., 2024). Notably, our data reveal specific orientation (150° and 60°) that maximize and minimum E_ROI_ ratios by 46.67 % and 50 %. While these orientation-dependent effects corroborate existing literature on single-target stimulation, our study extends this understanding through a novel dual-target paradigm. The significant maximum and minimum E-field ratio differences between anatomical and functional targets (*P* < 0.001) further substantiate the necessity of our dual-target computational model for precision neuromodulation. These findings provide valuable references for optimizing stimulation parameters in clinical practice. By optimizing coil orientation (e.g., selecting the perpendicular direction), precise activation of functional targets can be achieved while significantly reducing stimulation at anatomical targets. This refined control enhances the specificity and efficacy of TMS therapy. Particularly when adjusting stimulation intensity, a balance must be struck between activating functional targets and unintended responses at anatomical targets to achieve optimal therapeutic outcomes.

Although this study establishes the basic relationship between target methods and E-field characteristics, there are still some limitations. First, the findings are specific to figure-8 coils due to their focused E-field properties and directional dependence, and may not generalize to circular coils. Second, the lack of DTI data limits our comprehensive understanding of the role of WM anisotropy in E-field distribution and functional connectivity between targets. Future research could integrate fiber tracking technology to further assess the impact of structural networks on E-field distribution. Third, the study lacks randomized controlled trials comparing different targeting methods for the same subjects. Future studies should conduct more randomized controlled trials to compare the therapeutic effects of functional versus anatomical target localization. Fourth, age, gender and ethnicity are known E-field determinants (Ma et al., 2024), their systemic interactions with anatomical/functional targets require further investigation. Finally, the limited sample size may affect the generalizability of the results, and larger sample studies are needed in the future to validate these findings.

## 5 Conclusion

This study advances our understanding of the relationship between target localization methods (functional vs. anatomical), coil orientations, and resultant E-field characteristics in DLPC-TMS. Critically, we recommend employing orthogonal coil placement when targeting functional targets defined by individual fMRI data in AD patients. This approach is essential for minimizing off-target network activation and maximizing therapeutic efficacy by precisely engaging the intended functional circuits. These insights provide a valuable, evidence-based framework for optimizing TMS targeting protocols in both AD research and clinical applications, where precise neuromodulation is critical for cognitive outcomes. Future studies should validate these coil orientation effects in larger AD patients and further refine stimulation parameters based on individualized functional anatomy.

## CRediT authorship contribution statement

**Nianshuang Wu:** Writing – review & editing, Writing – original draft, Visualization, Supervision, Methodology, Investigation, Conceptualization. **Yuxuan Shao:** Visualization, Supervision, Methodology, Investigation, Data curation. **Zhen Wu:** Visualization, Supervision, Methodology, Investigation, Conceptualization. **Shuxiang Zhu:** Data curation. **Penghao Wang:** Conceptualization. **Ziyan Zhu:** Data curation. **Cheng Zhang:** Conceptualization. **Changzhe Wu:** Conceptualization. **Xiaolin Huo:** Conceptualization. **Hua Lin:** Visualization, Supervision, Project administration, Methodology, Investigation, Funding acquisition, Data curation, Conceptualization. **Guanghao Zhang:** Writing – review & editing, Writing – original draft, Visualization, Supervision, Project administration, Methodology, Investigation, Funding acquisition, Conceptualization.

## Funding

This research was funded by several projects. The 10.13039/501100012166National Key R&D Program of China supported this work with the project number 2022YFC2402200. The 10.13039/501100001809National Natural Science Foundation of China provided support through the project numbers 51977205, 52077209, 82471498, and 10.13039/501100001809NSFC - AF 82211530041. Additionally, the Capital's Funds for Health Improvement and Research also contributed to this work with the project number CFH2022 - 2 - 2014. Neither of the mentioned funders was involved in the conception and performance of the study.

## Declaration of Competing Interest

The authors declare that they have no known competing financial interests or personal relationships that could have appeared to influence the work reported in this paper.

## Data Availability

The datasets and code used or analyzed during the current study are available from the corresponding author on reasonable request.
