# Supplementary material for: Electric field variations across DLPFC targeting methods in TMS therapy for Alzheimer’s disease
Source: Neuroimage Clin. 2025 Jul 17;48:103847. doi: 10.1016/j.nicl.2025.103847 (PMC12305317; doi:10.1016/j.nicl.2025.103847)
Supplement: Supplementary Data 1 [file mmc1.docx]

**Suplementary Tables**

Table S1. Comparison of the dispersion degree of different targets in AD patients

| ANOVA | | | | LSD | | |  |
| --- | --- | --- | --- | --- | --- | --- | --- |
| *F* | *P* | | η^2^ | Category | *P* | |  |
| 37.60 | | < 0.001 | 0.46 | Func-Anat | | < 0.001 |  |
|  |  |  |  | Func-F3 | | < 0.001 |  |
|  |  |  |  | Anat-F3 | | 0.62 | |

Note: Functional target (Func), anatomical target (Anat), and F3 target (F3).

Table S2. Comparison of the inter - distance among three targets in AD patients

| ANOVA | | | | LSD | | |  |
| --- | --- | --- | --- | --- | --- | --- | --- |
| *F* | *P* | | η^2^ | Category | *P* | |  |
| 72.13 | | < 0.001 | 0.62 | Func-Anat | | 0.087 |  |
|  |  |  |  | Func-F3 | | < 0.001 |  |
|  |  |  |  | Anat-F3 | | < 0.001 | |

Note: 1. Distance between functional and anatomical targets (Func-Anat); 2. Distance between functional and F3 targets (Func-F3); 3. Distance between anatomical and F3 targets (Anat-F3).

Table S3. Comparison of the voxel values among three targets in AD patients

| ANOVA | | | | LSD | | |  |
| --- | --- | --- | --- | --- | --- | --- | --- |
| *F* | *P* | | η^2^ | Category | *P* | |  |
| 0.14 | | 0.87 | 0.003 | Func-Anat | | 0.65 |  |
|  |  |  |  | Func-F3 | | 0.64 |  |
|  |  |  |  | Anat-F3 | | 0.99 | |

Note: Functional target (Func), anatomical target (Anat), and F3 target (F3).

Table S4. E-field comparison directly below the coil in AD patients

| ANOVA | | | | LSD | | |  |  |
| --- | --- | --- | --- | --- | --- | --- | --- | --- |
| *F* | *P* | | η^2^ | Category | *P* | |  |  |
| E_ROI_ | | | | | | |  |  |
| 0.31 | | 0.73 | 0.007 | Func-Anat | | 0.57 |  |  |
|  |  |  |  | Func-F3 | | 0.45 |  |  |
|  |  |  |  | Anat-F3 | | 0.85 | |  |
| E_⊥_ | | | | | | | | |
| 0.23 | | 0.80 | 0.005 | Func-Anat | | 0.70 | | |
|  |  |  |  | Func-F3 | | 0.51 | | |
|  |  |  |  | Anat-F3 | | 0.78 | | |

Note: Functional target (Func), anatomical target (Anat), and F3 target (F3).

Table S5. E-field comparison and its neighbor

| ANOVA | | | | | LSD | | |  |  |
| --- | --- | --- | --- | --- | --- | --- | --- | --- | --- |
| *F* | *P* | | | η^2^ | Category | *P* | |  |  |
|  | | | Coil was aligned with the functional target for E_ROI_ | | | | |  |  |
| 35.37 | | < 0.001 | | 0.45 | Func-Anat | | < 0.001 |  |  |
|  |  |  |  |  | Func-F3 | | < 0.001 |  |  |
|  |  |  |  |  | Anat-F3 | | 0.34 | |  |
|  | | | Coil was aligned with the anatomical target for E_ROI_ | | | | | | |
| 30.15 | | < 0.001 | | 0.41 | Func-Anat | | < 0.001 | | |
|  |  |  |  |  | Func-F3 | | < 0.001 | | |
|  |  |  |  |  | Anat-F3 | | 0.67 | | |
|  | | | Coil was aligned with the F3 target for E_ROI_ | | | | | | |
| 17.75 | | < 0.001 | | 0.29 | Func-Anat | | < 0.001 | | |
|  |  |  |  |  | Func-F3 | | < 0.001 | | |
|  |  |  |  |  | Anat-F3 | | 0.88 | | |
|  | | | Coil was aligned with the functional target for E_⊥_ | | | | | | |
| 25.35 | | < 0.001 | | 0.37 | Func-Anat | | < 0.001 | | |
|  |  |  |  |  | Func-F3 | | < 0.001 | | |
|  |  |  |  |  | Anat-F3 | | 0.59 | | |
|  | | | Coil was aligned with the anatomical target for E_⊥_ | | | | | | |
| 16.67 | | < 0.001 | | 0.28 | Func-Anat | | < 0.001 | | |
|  |  |  |  |  | Func-F3 | | < 0.001 | | |
|  |  |  |  |  | Anat-F3 | | 0.62 | | |
|  | | | Coil was aligned with the F3 target for E_⊥_ | | | | | | |
| 9.06 | | < 0.001 | | 0.17 | Func-Anat | | < 0.001 | | |
|  |  |  |  |  | Func-F3 | | < 0.001 | | |
|  |  |  |  |  | Anat-F3 | | 0.99 | | |

Note: Functional target (Func), anatomical target (Anat), and F3 target (F3).

Table S6. Ratio of functional to anatomical E-field in AD patients

| E_ROI_ | | | | | | |
| --- | --- | --- | --- | --- | --- | --- |
|  | 0° | 30° | 60° | 90° | 120° | 150° |
| sub1 | 1.38 | 1.07 | 1.03 | 1.16 | 1.52 | 1.74 |
| sub2 | 1.55 | 1.24 | 1.23 | 1.39 | 1.71 | 1.95 |
| sub3 | 1.40 | 1.15 | 1.17 | 1.31 | 1.62 | 1.89 |
| sub4 | 1.49 | 1.37 | 1.29 | 1.36 | 1.81 | 1.69 |
| sub5 | 1.31 | 1.19 | 1.20 | 1.39 | 1.69 | 1.59 |
| sub6 | 1.65 | 1.30 | 1.17 | 1.21 | 1.80 | 2.34 |
| sub7 | 2.00 | 1.50 | 1.22 | 1.19 | 1.28 | 1.64 |
| sub8 | 1.29 | 1.27 | 1.18 | 1.10 | 1.06 | 1.16 |
| sub9 | 1.77 | 1.39 | 1.32 | 1.51 | 2.20 | 2.67 |
| sub10 | 1.04 | 1.13 | 1.24 | 1.26 | 1.08 | 1.00 |
| sub11 | 1.70 | 1.28 | 1.18 | 1.23 | 1.48 | 2.05 |
| sub12 | 1.49 | 1.38 | 1.26 | 1.13 | 1.11 | 1.32 |
| sub13 | 1.21 | 1.22 | 1.34 | 1.37 | 1.23 | 1.14 |
| sub14 | 1.43 | 1.35 | 1.41 | 1.99 | 2.48 | 1.68 |
| sub15 | 0.89 | 0.97 | 1.10 | 1.16 | 1.06 | 0.89 |
| sub16 | 1.22 | 1.12 | 1.10 | 1.17 | 1.24 | 1.25 |
| sub17 | 2.74 | 1.82 | 1.43 | 1.29 | 1.39 | 2.02 |
| sub18 | 1.87 | 1.52 | 1.48 | 1.55 | 1.96 | 2.68 |
| sub19 | 1.52 | 1.30 | 1.38 | 1.44 | 1.57 | 1.84 |
| sub20 | 1.24 | 1.19 | 1.07 | 0.99 | 1.01 | 1.14 |
| sub21 | 1.65 | 1.21 | 1.16 | 1.37 | 1.62 | 1.75 |
| sub22 | 1.53 | 1.32 | 1.25 | 1.34 | 1.44 | 1.55 |
| sub23 | 1.79 | 1.62 | 1.41 | 1.18 | 1.09 | 1.43 |
| sub24 | 2.37 | 1.57 | 1.51 | 1.62 | 1.79 | 2.40 |
| sub25 | 8.47 | 2.94 | 2.30 | 2.12 | 2.09 | 3.23 |
| sub26 | 1.34 | 1.16 | 1.11 | 1.34 | 2.98 | 2.35 |
| sub27 | 2.66 | 1.67 | 1.49 | 1.54 | 1.81 | 2.99 |
| sub28 | 1.38 | 1.29 | 1.26 | 1.34 | 1.56 | 1.56 |
| sub29 | 1.22 | 1.24 | 1.29 | 1.34 | 1.36 | 1.30 |
| sub30 | 3.62 | 1.85 | 1.41 | 1.45 | 1.67 | 2.46 |
| E_⊥_ | | | | | | |
| sub1 | 1.41 | 1.13 | 1.08 | 1.08 | 1.18 | 1.55 |
| sub2 | 1.13 | 0.98 | 1.16 | 1.75 | 2.10 | 1.91 |
| sub3 | 0.97 | 0.95 | 1.20 | 1.46 | 1.92 | 2.53 |
| sub4 | 1.71 | 1.47 | 1.32 | 1.24 | 1.89 | 2.10 |
| sub5 | 1.48 | 0.93 | 0.69 | 1.19 | 2.05 | 2.20 |
| sub6 | 1.96 | 1.46 | 1.13 | 1.28 | 2.34 | 2.67 |
| sub7 | 2.17 | 1.72 | 1.35 | 1.26 | 1.26 | 1.47 |
| sub8 | 1.40 | 1.62 | 1.42 | 1.03 | 0.77 | 1.02 |
| sub9 | 1.84 | 1.42 | 1.34 | 1.50 | 2.02 | 2.37 |
| sub10 | 1.00 | 1.02 | 1.05 | 1.07 | 0.95 | 0.95 |
| sub11 | 1.60 | 1.24 | 1.16 | 1.20 | 1.60 | 2.54 |
| sub12 | 1.57 | 1.25 | 1.13 | 1.11 | 1.26 | 2.01 |
| sub13 | 1.09 | 1.12 | 1.14 | 1.33 | 1.39 | 1.17 |
| sub14 | 1.22 | 0.95 | 0.84 | 1.55 | 2.58 | 1.58 |
| sub15 | 0.64 | 1.05 | 1.37 | 1.48 | 1.24 | 0.67 |
| sub16 | 0.91 | 1.03 | 1.65 | 1.91 | 1.81 | 1.38 |
| sub17 | 2.80 | 1.83 | 1.43 | 1.03 | 1.09 | 2.27 |
| sub18 | 2.21 | 1.54 | 1.37 | 1.50 | 2.05 | 2.99 |
| sub19 | 1.22 | 1.22 | 1.41 | 1.45 | 1.57 | 1.86 |
| sub20 | 1.80 | 1.48 | 1.09 | 0.82 | 0.88 | 1.42 |
| sub21 | 1.26 | 1.16 | 1.41 | 1.77 | 2.18 | 1.88 |
| sub22 | 1.52 | 1.39 | 1.31 | 1.54 | 1.71 | 1.82 |
| sub23 | 2.35 | 2.35 | 1.83 | 1.13 | 0.58 | 1.03 |
| sub24 | 2.44 | 1.29 | 1.30 | 1.95 | 2.69 | 2.97 |
| sub25 | 8.83 | 3.02 | 2.60 | 2.33 | 2.01 | 2.92 |
| sub26 | 1.31 | 0.80 | 0.76 | 1.38 | 4.35 | 2.83 |
| sub27 | 2.03 | 1.80 | 1.83 | 1.80 | 1.69 | 2.24 |
| sub28 | 1.57 | 1.27 | 1.05 | 0.99 | 1.41 | 1.73 |
| sub29 | 1.43 | 1.42 | 1.50 | 1.46 | 1.41 | 1.63 |
| sub30 | 5.84 | 2.06 | 1.44 | 1.40 | 1.72 | 2.79 |

**Section 1. Clinical characteristics**

This study is registered in the Chinese Clinical Trial Registry, number ChiCTR2200062564, which investigated precision-guided rTMS for AD using novel individualized localization technology.

1. Intervention protocol:

(1) aiTBS Delivery: 28 sessions over 14 days (2 sessions/day, 50-min interval) using Magstim Rapid² with figure-8 coil. Target: Left DLPFC (MNI: −38, 44, 26) neuronavigated via structural MRI (Brainsight system).

(2) Parameters: 80% resting motor threshold; 1,800 pulses/session (60 triplet bursts; 2-s train duration, 8-s cycling period; total 50,400 pulses).

2. Cognitive assessments: Mini-Mental State Examination (MMSE), Montreal Cognitive Assessment (MoCA), Face-word Stroop Task, Auditory Verbal Learning Test (AVLT) at baseline and post-treatment.

Clinical features and cognitive status (as reported for the 30 patients in the manuscript) are as follows:

**Table S7 Summary of the patients’ clinical characteristic data**

|  | before | | after |
| --- | --- | --- | --- |
| Gender (M:F) | | 11:19 | / |
| Age | | 68.70±6.86 |  |
| MMSE | | 25.96±2.23 | 27.96±2.47 ^b,^ *** |
| MoCA | | 21.86±4.07 | 23.83±4.51 ^b,^ *** |
| Face-word Stroop Task | | 5.79±4.03 | 8.51±4.22 ^a,^ *** |
| AVLT1 | | 5.09±2.34 | 6.97±2.86 ^b,^ *** |
| AVLT2 | | 6.94±2.59 | 8.87±3.25 ^a,^ *** |
| AVLT3 | | 8.15±3.07 | 9.94±3.51 ^a,^ *** |
| AVLT_delay | | 6.24±3.81 | 8.17±4.58 ^b,^ *** |
| AVLT_clue | | 7.85±3.91 | 8.90±4.21 ^a,^ * |
| AVLT_recognition | | 8.27±4.21 | 9.53±3.00 ^a,^ ** |

Note. Measurement data are described as the mean ± standard deviation. Statistical analyses before and after treatment were performed using the paired t-test (a) and Wilcoxon test (b). * *P*<0.05, ** *P*<0.01, *** *P*<0.005

Abbreviations: MMSE, Mini-Mental State Examination; MOCA, Montreal Cognitive Assessment; AVLT, Auditory Verbal Learning Test.

**Section 2. The results of 15 additional cases**

We additionally enrolled 15 patients (4 males and 11 females, ages: 55-76, 66.20 ± 6.90), with identical inclusion criteria and E-field simulation processing procedures. The results are as follows:

**Table S8 Distances and voxel values across different targets in 15 additional cases**

|  | Mean ± SD | | *t* | *P* | *d* | effect size |
| --- | --- | --- | --- | --- | --- | --- |
|  | functional targets | anatomical targets |  |  |  |  |
| radial distance (mm) | 12.60 ± 5.05 | 4.13 ± 1.38 | 7.22 | <0.001 | 2.29 | 0.75 |
| ROI brain volumes (mm^3^) | 1312.87 ± 671.96 | 1245.27 ± 472.80 | 0.32 | 0.75 | 0.12 | 0.059 |

Among them, inter-target spatial distances functional-anatomical 27.45 ± 10.31 mm. The mean SCD across patients was 13.36 ± 2.12 mm for functional targets, followed by 12.45 ± 1.72 mm for anatomical targets.

***This finding is consistent with the 30 cases reported in the main manuscript****, showing the highest dispersion in functional targets, which was 3.05 times that of anatomical targets. Volumes analysis revealed comparable ROI brain volumes between targets, with no statistically significant difference.*

**Table S9 Comparison of E-field across different targets at the same coil orientation in 15 additional cases**

| E-field | Mean ± SD | | *t* | *P* | *d* | effect size |
| --- | --- | --- | --- | --- | --- | --- |
|  | functional target | anatomical target |  |  |  |  |
| E_ROI_ | 0.73 ± 0.12 | 0.77 ± 0.12 | -0.56 | 0.58 | -0.33 | -0.16 |
| E_⊥_ | 0.40 ± 0.095 | 0.36 ± 0.059 | -1.04 | 0.30 | 0.51 | 0.25 |

***This finding is consistent with the 30 cases reported in the main manuscript****.* Although functional and anatomical targets differ in spatial localization, the E-field (E_ROI_, E_⊥_) beneath the coil shows no significant difference between the two target types.

**Table S10 E-field comparison and its neighbor in 15 additional cases**

| E-field | Mean ± SD | | *t* | *P* | *d* | effect size |
| --- | --- | --- | --- | --- | --- | --- |
|  | functional target | anatomical target |  |  |  |  |
| Coil was aligned with the functional target | | | | | | |
| E_ROI_ | 0.73 ± 0.12 | 0.50 ± 0.19 | 5.83 | < 0.001 | 1.45 | 0.59 |
| E_⊥_ | 0.40 ± 0.095 | 0.23 ± 0.092 | 4.96 | < 0.001 | 1.82 | 0.67 |
| Coil was aligned with the anatomical target | | | | | | |
| E_ROI_ | 0.57 ± 0.17 | 0.77 ± 0.12 | -3.82 | 0.001 | -1.36 | -0.56 |
| E_⊥_ | 0.28 ± 0.093 | 0.36 ± 0.059 | -2.69 | 0.012 | -1.03 | -0.46 |

***This finding is consistent with the 30 cases reported in the main manuscript****.* Regardless of which target the coil is aligned with, there is a significant difference between the E-field directly beneath the coil and that of adjacent targets.

**Table S11 The impact of orthogonal coil positioning on target sites in 15 additional cases**

| E-field | Mean ± SD | | *t* | *P* | *d* | effect size |
| --- | --- | --- | --- | --- | --- | --- |
|  | functional target | anatomical target |  |  |  |  |
| coil handle parallel to the LOI | | | | | | |
| E_ROI_ | 0.74 ± 0.12 | 0.55 ± 0.13 | 6.30 | <0.001 | 1.52 | 0.60 |
| E_⊥_ | 0.42 ± 0.084 | 0.26 ± 0.070 | 5.97 | <0.001 | 2.07 | 0.72 |
| coil handle perpendicular to the LOI | | | | | | |
| E_ROI_ | 0.74 ± 0.10 | 0.34 ± 0.21 | 6.68 | <0.001 | 2.43 | 0.77 |
| E_⊥_ | 0.32 ± 0.062 | 0.15 ± 0.091 | 5.65 | <0.001 | 2.18 | 0.74 |

***This finding is consistent with the 30 cases reported in the main manuscript****.* When the coil handle was oriented parallel to the LOI, the functional target exhibited E-field ratios of 1.48 for E_ROI_ and 1.62 for E_⊥_. Importantly, rotating the coil handle to a perpendicular orientation relative to the LOI maintained the functional target's efficacy while significantly reducing activation of the anatomical target. Specifically, the functional target E_ROI_ remained stable at 0.74 ± 0.10. In contrast, the anatomical target E_ROI_ showed a marked reduction to 0.34 ± 0.21, representing a 38.18% decrease compared to its value (0.55 ± 0.13) under parallel coil alignment.

The E-field ratio between the functional target and anatomical target varied with coil rotation angles, showing statistically significant differences (*P* < 0.05) between the observed maximum and minimum ratios. ***This finding is consistent with the 30 cases reported in the main manuscript****.* This result demonstrates that coil orientation can modulate the relative E-field intensities of dual targets. Therefore, adjusting coil orientation provides an effective approach for achieving differential neuromodulation—maintaining E-fields at functional targets while suppressing those at anatomical targets. This establishes crucial parametric guidance for optimizing personalized neuromodulation protocols.

The results regarding E-field ratios at various orientations for functional and anatomical targets are as follows:

As the coil rotates from its initial position, it is positioned above the functional target site. At different orientations, we measured the ratio of functional to anatomical E-field. For the analysis of E_ROI_, the maximum ratio occurs at 0° (6.67%), 30° (20.00%), 60° (33.33%), 90° (13.33%), 120° (20.00%), and 150° (6.67%). The minimum ratio is observed at 0° (20.00%), 60° (26.67%), 90° (26.67%), 120° (13.33%), and 150° (13.33%). A T-test reveals a significant difference between the maximum (2.83 ± 1.47) and minimum (1.52 ± 0.46) values (*t* = 3.29, *P* = 0.003, *d* = 1.20, effect size = 0.52).

Similarly, the ratio of E_⊥_ exhibits the following variations: the maximum ratio is observed at 0° (20.00%), 30° (6.67%), 60° (26.67%), 90° (13.33%),120° (20.00%), and 150° (13.33%). The minimum ratio occurs at 0° (26.67%), 30° (13.33%), 60° (40.00%), 90° (6.67%),120° (13.33%). Statistical analysis demonstrates a significant difference between the maximum (2.80 ± 1.34) and minimum (1.41 ± 0.57) values (*t* = 3.69, *P* = 0.001, *d* = 1.35, effect size = 0.56).

**Section 3. The results of healthy controls**

We included 30 age-matched HCs (13 males and 17 females, ages: 55-79, 67.17 ± 7.44) selected from the OpenNeuro (https://openneuro.org/datasets/ds005270/versions/1.0.0) public dataset (Rieck. et al., 2024), applying identical E-field simulation procedures as the AD group.

**Table S12. Distances and voxel values between different targets in HCs**

|  | Functional targets | Anatomical targets |
| --- | --- | --- |
| SCD (mm) | 14.17 ± 2.76 | 13.56 ± 2.00 |
| radial distance (mm) | 19.56 ± 7.03 | 3.70 ± 2.54 |
| ROI brain volumes (mm^3^) | 1322.63 ± 718.28 | 1323.17 ± 765.88 |

Among them, inter-target spatial distances functional-anatomical 27.25 ± 10.76 mm.

Regarding the comparison between HCs and AD patients, we found no significant differences in SCD. This aligns with existing literature since AD patients consisted of MCI stage patients (Lu et al., 2021). The detailed results show:

For functional targets: HCs: 14.17 ± 2.76 mm, AD patients: 14.76 ± 2.41 mm, *t* = -0.87, *P* = 0.39, *d* = -0.23, effect size = -0.11;

For anatomical targets: HCs: 13.56 ± 2.00 mm, AD patients: 12.75 ± 1.62 mm, t = 1.72, P = 0.091, *d* = 0.45, effect size = 0.22.

Notably, functional targets demonstrated significantly greater spatial variability than anatomical targets in HCs (mean radial distance: 19.56 ± 7.03 mm vs. 3.70 ± 2.54 mm; *t* = 17.45, *P* < 0.001, *d* = 3.00, effect size = 0.83), consistent with the result in AD patients. Functional targets showed comparable ROI brain volumes to anatomical targets in both groups (1322.63 ± 718.28 mm³ vs. 1323.17 ± 765.88 mm³; *t* = -0.003, *P* = 0.99, *d* = -0.00073, effect size = -0.00036), aligning with the result in AD patients.

**Table S13 Comparison of E-field across different targets beneath the coil in HCs**

| E-field | Mean ± SD | | *t* | *P* | *d* | effect size |
| --- | --- | --- | --- | --- | --- | --- |
|  | functional target | anatomical target |  |  |  |  |
| E_ROI_ | 1.02 ± 0.58 | 0.95 ± 0.52 | 0.51 | 0.62 | 0.13 | 0.063 |
| E_⊥_ | 0.48 ± 0.35 | 0.43 ± 0.25 | 0.42 | 0.58 | 0.16 | 0.082 |

**Table S14 E-field comparison and its neighbor in HCs**

| E-field | Mean ± SD | | *t* | *P* | *d* | effect size |
| --- | --- | --- | --- | --- | --- | --- |
|  | functional target | anatomical target |  |  |  |  |
| Coil was aligned with the functional target | | | | | | |
| E_ROI_ | 1.02 ± 0.58 | 0.64 ± 0.37 | 3.03 | 0.004 | 0.78 | 0.36 |
| E_⊥_ | 0.48 ± 0.35 | 0.29 ± 0.17 | 2.60 | 0.012 | 0.69 | 0.33 |
| Coil was aligned with the anatomical target | | | | | | |
| EROI | 0.72 ± 0.55 | 0.95 ± 0.52 | -1.66 | 0.10 | -0.43 | -0.21 |
| E_⊥_ | 0.33 ± 0.31 | 0.43 ± 0.25 | -1.38 | 0.17 | -0.36 | -0.17 |

**Table S15 The impact of orthogonal coil positioning on target sites in HCs**

| E-field | Mean ± SD | | *t* | *P* | *d* | effect size |
| --- | --- | --- | --- | --- | --- | --- |
|  | functional target | anatomical target |  |  |  |  |
| coil handle parallel to the LOI | | | | | | |
| E_ROI_ | 0.98 ± 0.59 | 0.70 ± 0.34 | 2.27 | 0.027 | 0.58 | 0.28 |
| E_⊥_ | 0.46 ± 0.35 | 0.32 ± 0.16 | 1.98 | 0.052 | 0.51 | 0.25 |
| coil handle perpendicular to the LOI | | | | | | |
| E_ROI_ | 0.94 ± 0.39 | 0.43 ± 0.27 | 5.94 | < 0.001 | 1.52 | 0.61 |
| E_⊥_ | 0.43 ± 0.15 | 0.19 ± 0.12 | 7.01 | < 0.001 | 1.77 | 0.66 |

**The results regarding E-field ratios at various orientations for functional and anatomical targets are as follows:**

As the coil rotates from its initial position, it is positioned above the functional target site. At different orientations, we measured the ratio of functional to anatomical E-field. For the analysis of E_ROI_, the maximum ratio occurs at 0° (3.33%), 30° (10.00%), 60° (23.33%), 90° (26.67%), 120° (20.00%), and 150° (16.67%). The minimum ratio is observed at 0° (10.00%), 30° (16.67%), 60° (20.00%), 90° (13.33%), 120° (16.67%), and 150° (23.33%). A T-test reveals a significant difference between the maximum (3.27 ± 2.16) and minimum (1.41 ± 0.34) values (*t* = 4.67, *P* < 0.001, *d* = 1.20, effect size = 0.52).

Similarly, the ratio of E_⊥_ exhibits the following variations: the maximum ratio is observed at 30° (16.67%), 60° (16.67%), 90° (16.67%),120° (23.33%), and 150° (26.67%). The minimum ratio occurs at 0° (16.67%), 30° (16.67%), 60° (13.33%), 90° (13.33%), 120° (20.00%), 150° (20.00%). Statistical analysis demonstrates a significant difference between the maximum (3.67 ± 2.49) and minimum (1.26 ± 0.46) values (*t* = 5.21, *P* < 0.001, *d* = 1.35, effect size = 0.56).

Voxel-based morphometry (VBM) analysis comparing 30 AD patients and 30 HCs revealed gray matter atrophy in the following regions, which is consistent with previous studies (Chen et al., 2024; Patterson et al., 2019):

**Table S16 VBM analysis comparing 30 AD patients and 30 HCs**

| Regions | No. of voxels | MNI coordinates  (x, y, z) | Clusters’ breakdown | (*t*) *P* | *d* | effect size |
| --- | --- | --- | --- | --- | --- | --- |
| Cluster 1 | 1477 | -27, -78, 53 | Parietal Lobe, Parietal_Inf_L,  Parietal_Sup_L | (7.24) <0.001 | 1.70 | 0.65 |
| Cluster 2 | 794 | -20, -68, 35 | Occipital Lobe, Occipital_Sup_L | (5.07) <0.001 | 1.18 | 0.51 |
| Cluster 3 | 643 | 15, -38, 32 | Cingulate Gyrus, Cingulum_Mid_R, Frontal Lobe | (4.67) <0.001 | 1.10 | 0.48 |
| Cluster 4 | 628 | -63, -59, 21 | Temporal Lobe, Temporal_Sup_L | (4.77) <0.001 | 1.33 | 0.55 |

**Section 4. MRI processing workflow**

(1) Functional target identification tool: Functional targets were determined using the TMStarget software (https://github.com/jigongjun/Neuroimaging-and-neuromodulation).

(2) Head motion artifact control procedure: Initial Screening: Participants exhibiting excessive head motion (>2 mm or 2°) were excluded. Subject-Specific Processing: For each participant's fMRI data, motion correction was performed using the TMStarget software. Furthermore, fine adjustments were made within the TMStarget software based on the individual characteristics of each participant's data.

(3) Nuisance regression variables: During preprocessing regression, the following parameters were selected and regressed out: Head Motion (HM) parameters, Cerebrospinal Fluid (CSF) signals, Global Signal (GS).

**Section 5. The correlation between SCD and E-Field**

After controlling for age, we further examined the relationship between SCD and E-field (see Table S17).

**Table S17 The correlation between SCD and E-Field**

|  | *r* | *P* |
| --- | --- | --- |
| Functional target | | |
| E_ROI_ | 0.48 | 0.009 |
| E_⊥_ | 0.36 | 0.053 |
| Anatomical target | | |
| E_ROI_ | 0.40 | 0.032 |
| E_⊥_ | 0.47 | 0.011 |


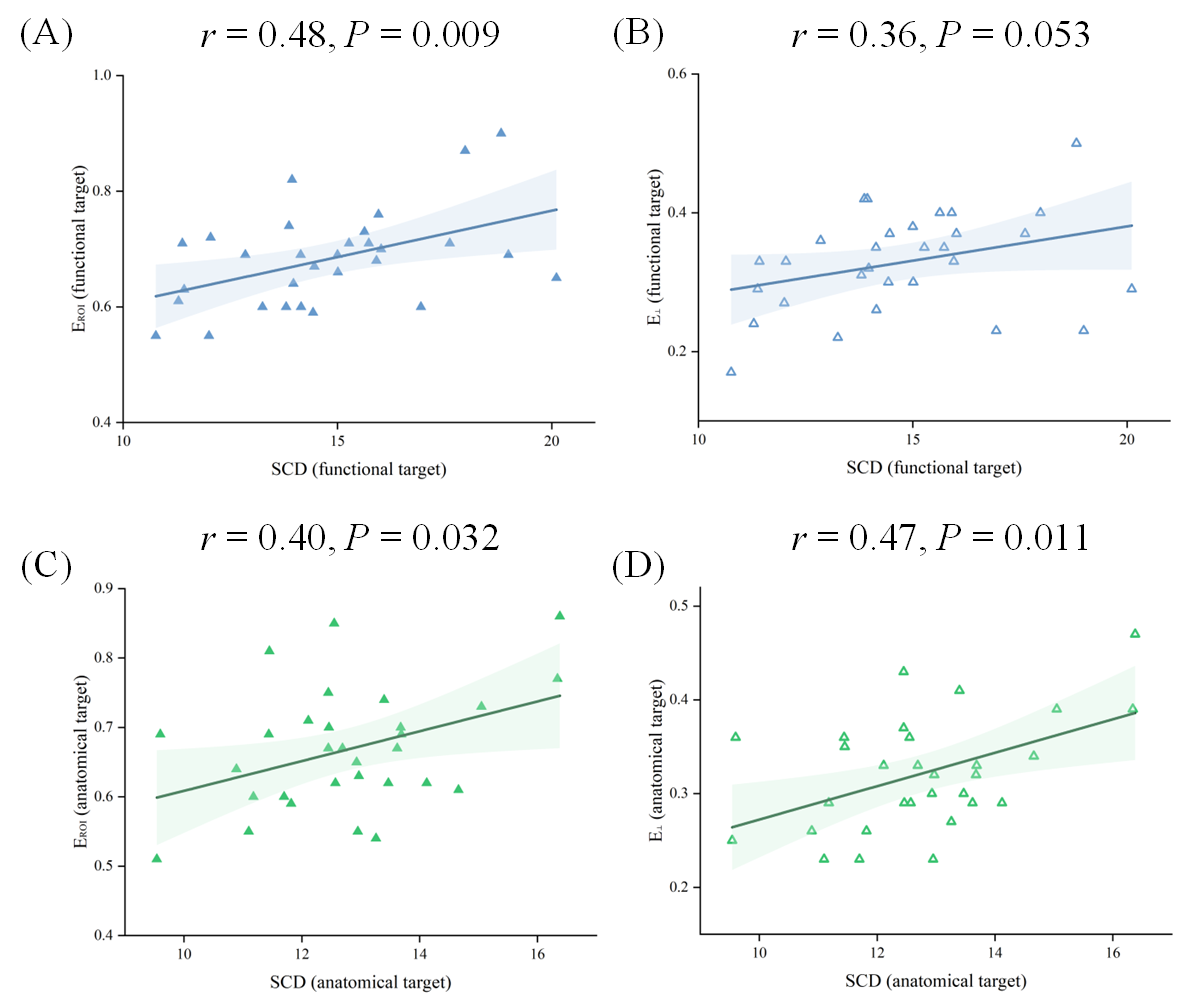


Fig. S1. Controlling for age as a covariate, the correlation between SCD and E-fields.

**Reference**

Chen, H., Yang, A., Huang, W., et al., 2024. Associations of quantitative susceptibility mapping with cortical atrophy and brain connectome in Alzheimer's disease: A multi-parametric study. Neuroimage. 290, 120555. <https://doi.org/10.1016/j.neuroimage.2024.120555>.

Lu, H., Li, J., Zhang, L., et al., 2021. Dynamic changes of region-specific cortical features and scalp-to-cortex distance: implications for transcranial current stimulation modeling. J Neuroeng Rehabil. 18(1), 2. <https://doi.org/10.1186/s12984-020-00764-5>.

Patterson, L., Firbank, M. J., Colloby, S. J., et al., 2019. Neuropathological Changes in Dementia With Lewy Bodies and the Cingulate Island Sign. Journal of Neuropathology and Experimental Neurology. 78(8), 717-724. <https://doi.org/10.1093/jnen/nlz047>.

Rieck., J. R., Baracchini., G., DeSouza., B., et al., 2024. BOLD variability during cognitive control for an adult lifespan sample. OpenNeuro. [Dataset] https://doi.org/doi:10.18112/openneuro.ds005270.v1.0.0.
